# Case of Labor Complicated with Prolapsus of the Vagina

**Published:** 1857-02

**Authors:** L. C. White

**Affiliations:** Savannah, Ill.


ARTICLE III.—*Case of Labor Complicated with Prolapsus of the Vagina.* By L. C. WHITE, M.D. of Savannah, Ill.

Having had a case recently that presented some points of interest, I have thought it might not be amiss to give its details publicity.

On the 25th Sept. last, I was called upon to visit Mrs. H. about seven miles out of town, in her eighth confinement. On making an examination, I found a tumor, anteriorly to the vulva, representing the circumference of the vaginal canal, hard and resisting, and of dimensions corresponding to the size of a perforated placenta. The labor had progressed somewhat; os uteri dilated; the head passing the superior strait in a normal position. Resuming the examination, I became convinced that it was not a tumefied condition of the labiæ, but a relaxation of the walls of the vagina—in other words a prolapsus of that organ.

To reduce the tumor seemed impossible, but thinking it might not interfere materially with the progress of the case, I concluded to wait patiently for events.

I will here remark, *en passant*, that this same relaxation had existed (so my patient informed me) in her last two or three confinements, and that it had been down for two weeks previous to the present time.

Waiting about six hours, during which time the pains were exceedingly feeble, and feeling anxious to terminate the delivery as soon as possible, I administered twenty grains of the secale, in infusion, and repeated twice at intervals of twenty minutes. Its administration produced its effect, but the prolapsus, despite my efforts, increased upon me.

At four o'clock A.M. sixteen hours after my arrival, the head presented at the inferior strait, but further progress seemed mechanically impossible owing to the increased prolapsus and accompanying tumefaction. I had previously urged the necessity of *turning*, but it was looked upon with decided disfavor. I now informed the husband that the child must be sacrificed in order to save the life of the mother. I then at once dispatched a messenger for my instruments, and at the same time summoned my friend Dr. Woodruff, of Savannah, wishing to have the aid of counsel in so unusual, so unique a case.

As the pains were at this time powerfully propulsive, without the remotest possibility of doing any good, I gave a full dose of sulph. morphia, which had the effect to produce an entire cessation, so that when Dr. W. arrived, four hours thereafter, the head had receded, and I had been enabled to reduce the tumor to the condition in which I found it. Turning was still objected to, and there was so much tumefaction externally that it was thought impossible to apply the forceps. Shortly the pains returned and the head presented, preceded by the walls of the vagina as before. The tumefaction was enormous completely filling up the passage, and the head could not pass by any effort of nature, I, therefore, with a cutting instrument, reduced the diameter of the head, and with the aid of the blunt hook succeeded in accomplishing delivery—a very laborious and difficult operation indeed, occupying about half an hour in performing it. The tumor externally was large enough to weigh four pounds, but from the pressure against the pubes was in a paralyzed condition.

Being taken ill myself, the subsequent treatment of the case devolved upon Dr. Woodruff, who informs me that the sloughing was somewhat extensive, and he thinks there must be immense cicatrices within the vaginal canal, which will probably preclude the same amount of relaxation hereafter. But the remedy may prove to be as bad as the original disease; at least I should think so. I should say that Dr. Woodruff succeeded in reducing a large portion of the prolapsed organ.

Now, is this a new disease, or is it not? I cannot find in any of the standard works on obstetrics an allusion to such a malady as prolapsus of the vagina, or even relaxation of it. I do not, therefore, think that such a case has been provided for, or even anticipated by obstetrical writers.

But I do not claim to be the discoverer of a new disease in this connection, or to have produced it. This honor, if honor it be, belongs to another one of the genus homo. Mrs. H. the lady in question, was attended in a confinement about five years since by an individual of the Thompsonian school, ycleped Eclectic. Being summoned in attendance, he promptly responded with about two quarts of tr. lobelia, and a ten ounce syringe. Suffice it, that after keeping his patient under the influence of his 'remedy' about twenty-four hours, the system became so much relaxed that the vagina *came down*, and she was delivered in great peril. The relaxation has continued ever since—and it had not existed before. What a commentary upon quackery! and what a disgrace to the Medical Profession it is, that such facilities exist for unqualified and unscrupulous men to enter its precincts and dub themselves with its honors.

*Savannah, Jan.* 1857.

